# Previous Lumbar Spine Surgery Decreases the Therapeutic Efficacy of Dorsal Root Ganglion Pulsed Radiofrequency in Patients with Chronic Lumbosacral Radicular Pain

**DOI:** 10.3390/jpm13071054

**Published:** 2023-06-27

**Authors:** Jiri Jandura, Milan Vajda, Roman Kostysyn, Jiri Vanasek, Eva Cermakova, Jan Zizka, Pavel Ryska

**Affiliations:** 1 Department of Diagnostic Radiology, University Hospital Hradec Kralove, Sokolska 581, 50005 Hradec Kralove, Czech Republic; jiri.jandura@fnhk.cz (J.J.); milan.vajda@fnhk.cz (M.V.); jiri.vanasek@fnhk.cz (J.V.); 2 Department of Diagnostic Radiology, Faculty of Medicine in Hradec Kralove, Charles University, Simkova 870, 50003 Hradec Kralove, Czech Republic; roman.kostysyn@fnhk.cz; 3 Department of Neurosurgery, University Hospital Hradec Kralove, Sokolska 581, 50005 Hradec Kralove, Czech Republic; 4 Department of Medical Biophysics, Faculty of Medicine in Hradec Kralove, Charles University, Simkova 870, 50003 Hradec Kralove, Czech Republic; cermakovae@lfhk.cuni.cz; 5 Department of Imaging and Functional Medicine, University of Umea Daniel Naezéns väg, 90737 Umea, Sweden; jan-zizka@seznam.cz

**Keywords:** pain management, pulsed radiofrequency, dorsal root ganglion, spine, surgery

## Abstract

Chronic lumbosacral radicular pain (CLRP) as a possible adverse consequence of lumbar spine surgery represents a serious medical challenge. Pulsed radiofrequency of dorsal root ganglion (PRF–DRG) treatment is known to be effective in alleviating CLRP. This retrospective study compares the efficacy of a single CT-guided PRF–DRG procedure in the treatment of unilateral CLRP between patients without (non-PSS) and with (PSS) previous lumbar spine surgery. Non-PSS and PSS groups included 30 and 20 patients, respectively. Outcomes (pain intensity and disability) were evaluated by means of the visual analog scale (VAS) and Oswestry disability index (ODI) immediately after the procedure (VAS), as well as three and six months after the procedure, respectively. Non-PSS group showed a significant (*p* ˂ 0.001) decrease of VAS (median) at all follow-up intervals (from 6 to 4; 4; 4.5 points, respectively). The PSS group showed a significant yet transient VAS (median) decrease (from 6 to 5 points) immediately after the procedure only (*p* < 0.001). The decrease of VAS was more pronounced in the non-PSS group after three and six months (*p* = 0.0054 and 0.011, respectively) in intergroup comparison. A relative decrease of VAS ≥ 50% during follow-up was achieved in 40%; 43.3%; 26.7% (non-PSS), and 25%; 5%; 0% (PSS) of patients. ODI (median) significantly decreased in the non-PSS group (from 21.5 to 18 points) at three and six months (*p* = 0.014 and 0.021, respectively). In conclusion, previous lumbar spine surgery decreases the therapeutic efficacy of PRF–DRG procedure in CLRP patients.

## 1. Introduction

Lumbosacral radicular pain (LRP) is a common clinical finding with a statistical prevalence ranging from 9.9% to 25% in the general population [1]. Whereas cervical radicular pain affects approximately 1 in 1000 adults, the prevalence of LRP reported in the literature is at least 10 times higher [2,3]. Dworkin et al. stated that LRP is probably the most common type of neuropathic pain [4]. Patients with LRP may experience reduced functional ability and quality of life [5]. About three-quarters of patients with acute LRP can recover considerably within a few months; however, the prognosis of persistent chronic radicular pain is not favorable [6,7]. Despite its high prevalence and significant impact on quality of life, the optimal conservative treatment for patients with radicular pain is not known [8]. LRP is defined as pain perceived as arising in the lower limb. It is caused by ectopic activation of nociceptive afferent fibers in a spinal nerve or its roots, or other neuropathic mechanisms resulting, for example, from disc protrusion, spinal stenosis, facet joint hypertrophy, or fibrosis after lumbar spine surgery. The pathophysiology of chronic lumbosacral radicular pain (CLRP) involves mechanical, inflammatory, and immunological factors that affect the function of the dorsal root ganglion (DRG), which most likely is the site of the origin of ectopic impulses in patients with radicular pain and the primary target for neuromodulator pain treatment, such as pulsed radiofrequency (PRF) [9].

Minimally invasive PRF treatment introduced by Sluijter in 1998 is known to be safe and effective in alleviating radicular pain. It delivers an electromagnetic field and heat bursts to the target nerves or tissues without macroscopic structural damage to these structures [10]. PRF was developed, in part, as a less destructive alternative to continuous radiofrequency (CRF), which has been in use since the mid-1970s. PRF offers the advantage of pain control without the tissue destruction and painful sequelae associated with CRF [11]. Radiofrequency (RF) is an alternating electric field with an oscillating frequency of 500.000 Hz. If the resulting current flows through a percutaneously introduced electrode, heat will be produced around the electrode because the body tissue acts as a resistor. The method of PRF is based on the concept that the production of heat has been a by-product of RF treatment and that the clinical effect is due to exposure to the electric field. In PRF, the generator output is interrupted to allow for the elimination of heat in the silent period [12]. Suggested pain-reducing mechanisms of PRF–DRG treatment include a decrease of microglial activity, reduction of proinflammatory cytokines, increase in the levels of endogenous opioid precursor messenger RNA and the corresponding opioid peptide, activation of the pain-inhibitory mechanism, inhibition of the excitatory nociceptive C-fibers and microscopic damage of the nociceptive nerve [13]. Minimally invasive PRF–DRG treatment requires the correct placement of the RF cannula next to the DRG. This is usually ensured by imaging guidance utilizing CT or fluoroscopy [14].

CLRP may develop as an adverse consequence of lumbar spine surgery. Therefore, it can also be considered as one of the manifestations of the failed back surgery syndrome (FBSS). The term FBSS embraces a constellation of conditions that describes persistent or recurring low back pain, with or without sciatica following one or more spine surgeries [15]. The overall failure rate of lumbar spine surgery in the United States is estimated to range from 10% to 46%, and despite advances in technology and surgical technique, the absolute number of patients developing FBSS can be expected to increase continually [16]. For example, in the United States, the number of spinal fusion surgeries increased from 174,233 in 1998 to 413,171 in 2008 [17]. Treatment of radicular pain persisting after spinal surgery usually requires a multidisciplinary approach [15]. Minimally invasive procedures are proposed as a therapeutic option to relieve the radicular pain associated with FBSS [18], which might result in further increase of indications for PRF–DRG treatment. The evidence concerning the therapeutic efficacy of PRF–DRG treatment of CLRP after spinal surgery is limited and somewhat contradictory.

The rationale for this study is to elucidate the potential benefits of minimally invasive PRF–DRG procedure in the treatment of CLRP in subjects who either have or have not undergone previous lumbar spine surgery. Minimally invasive methods (including PRF–DRG) are commonly indicated as a treatment option for patients with CLRP. Although PRF–DRG itself can be considered a very safe modality, its therapeutic benefits should outweigh any potential risks arising from the nature of the minimally invasive treatment. Similarly, the financial efficiency aspect of this treatment can also be important. From these perspectives, we consider a comparison of the efficacy of PRF–DRG between the compared patient groups to be meaningful.

## 2. Materials and Methods

### 2.1. Study Design and Patient Selection

This retrospective monocentric observational study is related to the previously published prospective trial evaluating the effectiveness of the three minimally invasive methods of CLRP treatment (pulsed radiofrequency, oxygen–ozone therapy, and epidural steroid injections) in patients with CLRP [19]. This study was conducted in the Department of Radiology of the University Hospital Hradec Kralove in the Czech Republic. Eligible patients suffered from unilateral CLRP at L5 or S1 level. Each patient enrolled in this study underwent a single PRF–DRG therapeutic intervention and a 6-month post-treatment follow-up. All procedures were performed in the period between 11/2015 and 12/2017.

**Figure 1:**
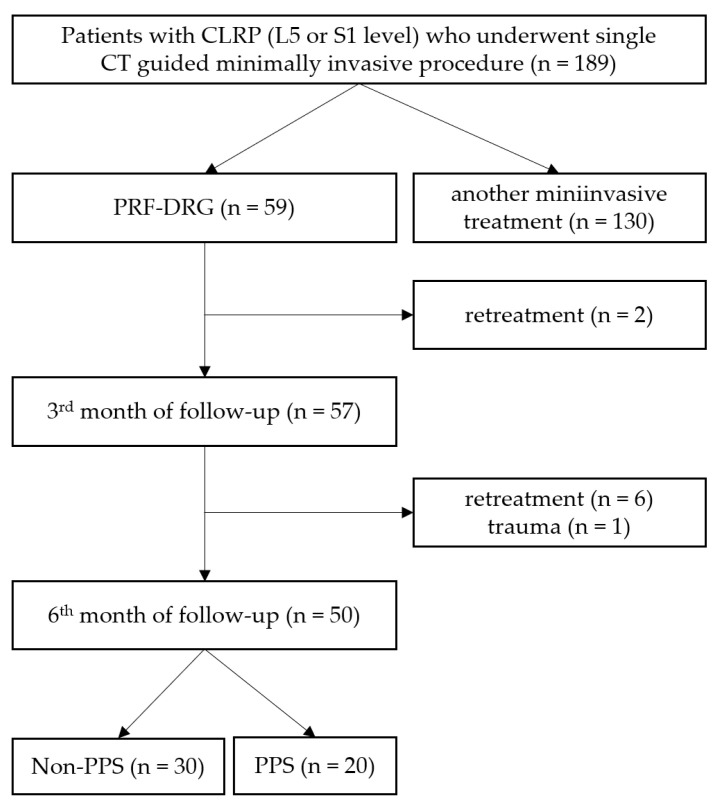
Patients’ enrollment flowchart. CLRP, chronic lumbosacral radicular pain; PRF–DRG, pulsed radiofrequency of dorsal root ganglion; non-PSS and PSS, groups of patients without and with a history of previous lumbar spine surgery.

This study was approved by the Institutional Ethics Committee and was conducted in accordance with the Declaration of Helsinki. All patients signed an informed consent to the treatment and agreed with the post-treatment follow-up. A total of 50 patients who underwent single PRF–DRG procedure and met the inclusion criteria were divided into non-PSS (no previous spinal surgery; *n* = 30) or PSS (positive history of previous spinal surgery; *n* = 20) groups (Figure 1).

The inclusion criteria were as follows: non-progressive chronic (at least 3 months lasting) unilateral mono-segmental radicular pain in the dermatome of L5 or S1; not satisfactorily responding to the conservative therapy; VAS score ≥ 4/10; spinal pathology (e.g., spinal stenosis) on MRI or CT; age ≥ 18 years; and completed follow-up (6 months). The exclusion criteria were as follows: lower extremity paresis or paralysis; sphincter insufficiency; infection; oncologic disease; relevant internal comorbidity (e.g., diabetes mellitus); hemorrhagic diathesis or anticoagulation (INR > 1.2); allergy to the materials and substances used; implanted cardiac pacemaker or spinal cord stimulating device; and follow-up not completed (i.e., <6 months).

### 2.2. Settings and Equipment

PRF–DRG treatment procedures were performed on an outpatient basis by two radiologists experienced in the minimally invasive treatment of CLRP. CT scanner Somatom Definition AS+ (Siemens, Erlangen, Germany) was used for guidance of the RF cannula utilizing low-dose radiation protocol. PRF–DRG equipment consisted of RF generator RFG˗1B (Cosman Medical, Burlington, MA, USA), a dispersive and thermocouple electrode, and a 22G SMK RF cannula: length 100 mm (150 mm), active tip 5 mm (NeuroTherm, Amsterdam, The Netherlands).

### 2.3. PRF–DRG Procedure

**Figure 2:**
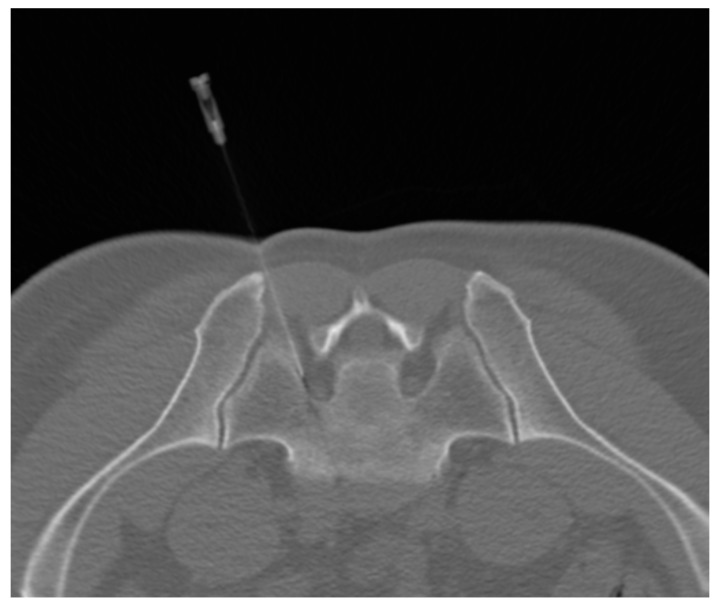
A transverse low-dose CT scan demonstrates the positioning of the radiofrequency cannula adjacent to the dorsal root ganglion of the left-sided S1 nerve root.

The patient was prone on the CT table, and the dispersive electrode was attached to the patient’s thigh contralateral to the symptomatic (treated) side. The RF cannula was stepwise introduced to the neuroforamen, and each positioning step was CT verified (Figure 2). Then, the stylet of the RF cannula was replaced by the thermocouple electrode. The distance between the electrode tip and the target DRG was subsequently verified by sensitive and motor nerve stimulation and corrected in case of need.

Optimally, the patient confirmed a change in perception (e.g., tingling) to the sensitive stimulation at the frequency of 50 Hz, within the voltage range U = 0.3–0.5 V. Similarly, positive reaction to the motor stimulation was expected at the frequency of 2 Hz, within the voltage range U = 0.5–0.7 V. The PRF–DRG treatment was set as follows: pulse width = 20 ms, f = 2 Hz, U = 45 V, Z ˂ 500 Ω, duration of PRF activity 2 × 120 s. The temperature at the electrode tip did not exceed 42 °C during the procedure. Patients were repeatedly checked for possible worsening pain or other discomfort during the treatment.

Eventually, the patients were strongly recommended to refrain from physical activities for the next two days after the treatment.

### 2.4. Outcome Evaluation

A visual analog scale (VAS; 0–10 points ranging from no pain to the most severe pain) was used to quantify the changes in radicular pain intensity over time. In addition, a proportion of relative VAS reduction ≥ 50% from the initial value was assessed. The patients’ disability was quantified by means of the Oswestry disability index (ODI; 0–50 points ranging from minimal disability to bedridden/exaggerating their symptoms) utilizing the Oswestry disability questionnaire. Acquired VAS and ODI data reflected the patient’s condition before the therapy and at the end of the 3rd and 6th month of follow-up, respectively. The early post-treatment VAS value was obtained from an interview with the patient immediately after the PRF–DRG procedure had been applied.

### 2.5. Statistical Analysis

Post hoc power analysis for both compared groups was made utilizing the paired sample T-test, Mann–Whitney, and Wilcoxon test (unpaired and paired comparison). Power (1 − β) was set at 80%, α at 5%. The calculated power values varied but reached a value of 0.99 in both groups. Calculated power Median was chosen for the presentation of the data due to the non-normal distribution of the descriptive data (age and body mass index (BMI)) and due to semiquantitative characteristics of VAS and ODI. The variability of the semiquantitative data was defined by the interquartile range. Intergroup comparison of the descriptive data was performed by Kolmogorov–Smirnov test (age, BMI) and Fisher’s exact test (sex). The Mann–Whitney U test was used for VAS and ODI intergroup comparison in pre- and post-treatment follow-up evaluations. The evolution of VAS and ODI over time (difference between pre- and post-treatment data) was assessed within each group separately by using a non-parametric analysis of variance (Dunnett–Friedman test). Fisher’s exact test was used for the intergroup comparison of relative VAS reduction ≥ 50% during the follow-up. The calculations were performed by NCSS 2019 software, version 19.0.4. (NCSS, LCC, Kaysville, UT, USA). The level of statistical significance was set to *p* < 0.05.

## 3. Results

### 3.1. Patients’ Characteristics

Patient characteristics and numbers of DRGs treated are summarized in Table 1. Non-PSS group consisted of 12 males and 18 females (age range 23–72 years). The PSS group consisted of 9 males and 11 females (age range 36–68 years). Regarding patients’ characteristics, a significant difference (*p* = 0.043) was found in BMI, which was higher in the non-PSS group. No significant intergroup difference was found in other descriptive data (age, sex, number of treated DRGs). Characteristics of spine surgery in the PSS group are shown in Table 2. The majority of lumbar spine surgeries were represented by non-fusion procedures, mostly by intervertebral disc hernia extractions utilizing partial hemilaminectomy (in 14 patients). Foraminotomies were also performed, both in fusion and non-fusion surgeries. In one patient, the intervertebral disc was replaced with an implant. One patient underwent repeat surgery (3x).

### 3.2. Outcome Data

#### 3.2.1. Development of Pain Intensity (VAS)

**Figure 3:**
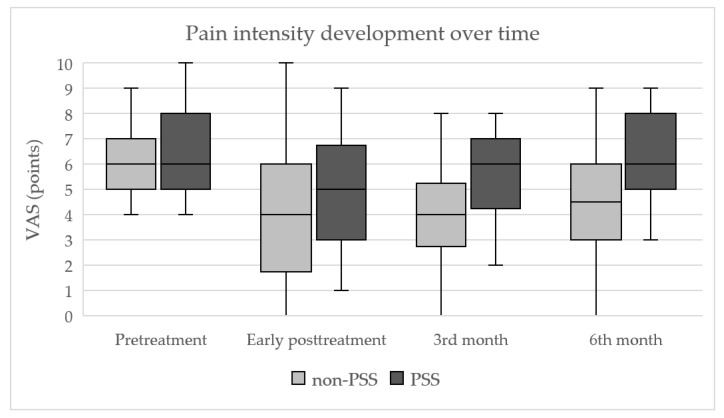
Development of pain intensity during the follow-up, quantified by visual analog scale. Box plots indicate the median (horizontal line) and interquartile range (box); whiskers indicate maximal and minimal values. VAS, visual analog scale; non-PSS and PSS, groups of patients without and with a history of previous lumbar spine surgery.

Follow-up in the post-treatment periods showed significant differences in pain reduction (VAS) between the compared groups, in favor of the non-PSS group. The median value of pre-treatment VAS was 6 points in both groups, without significant intergroup differences (*p* = 0.65). In the non-PSS group, the median VAS decreased from 6 to 4 points immediately after the procedure and remained unchanged until the third post-treatment month. Subsequently, the median VAS increased to 4.5 points in the sixth post-treatment month. The differences between pre- and post-treatment VAS within the non-PSS group proved to be statistically significant at all follow-up time points (all *p*-values ˂ 0.001). In the PSS group, the median VAS decreased from 6 to 5 points immediately after the procedure and returned to the pre-treatment level (6 points) during the follow-up at both three and six months. The significant difference between pre- and post-treatment VAS values in the PSS group was found for the early post-treatment timepoint only (*p* < 0.001). Intergroup comparison showed that the post-treatment decrease of the VAS values at both three and six months was significantly more expressed in the non-PSS group (*p* = 0.0054 and 0.011, respectively). The development of pain intensity over time is shown in Figure 3.

#### 3.2.2. Relative Decrease of VAS ≥ 50%

**Figure 4:**
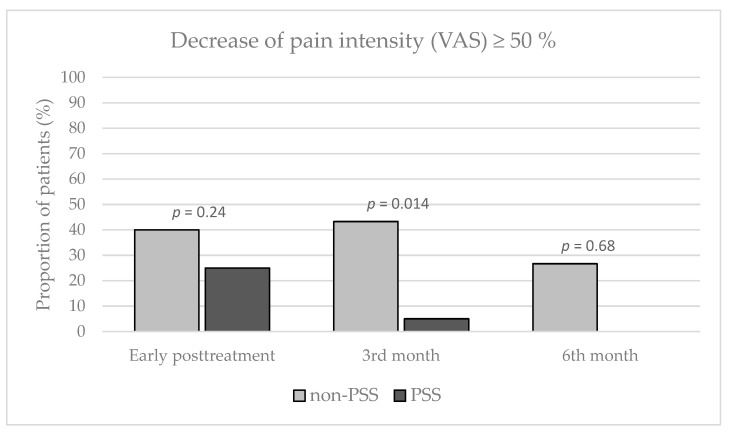
Decrease of pain intensity ≥ 50% during the follow-up, quantified by visual analog scale. VAS, visual analog scale; non-PSS and PSS, groups of patients without and with a history of previous lumbar spine surgery; *p*, *p*-value indicating the intergroup difference (utilizing Fisher’s exact test).

The relative decrease of VAS ≥ 50% within the follow-up time points (immediately after treatment, at three and six months after treatment, respectively) was observed in 12 (40%), 13 (43.3%), and 8 (26.7%) patients in the non-PSS group; alternatively in 5 (25%), 1 (5%) and 0 (0%) patients in the PSS group. The intergroup difference was found significant three months after treatment (*p* = 0.014) in favor of the non-PSS group (Figure 4).

#### 3.2.3. Development of Patients’ Disability (ODI)

**Figure 5:**
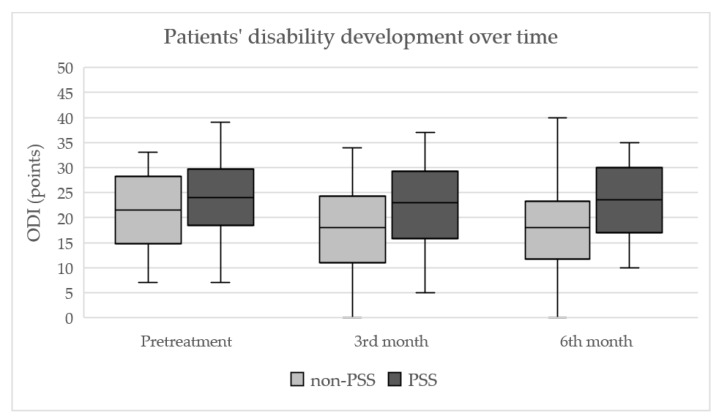
Development of patients’ disability during the follow-up, rated by the Oswestry disability index. Box plots indicate the median (horizontal line) and interquartile range (box); whiskers indicate maximal and minimal values. ODI, Oswestry disability index; non-PSS and PSS, groups of patients without and with a history of previous lumbar spine surgery.

The development of ODI over time is shown in Figure 5. Median ODI values (pre-treatment, at three and six months after treatment) were as follows: 21.5; 18; 18 points in the non-PSS group and 24; 23; 23.5 points in the PSS group, respectively. Post-treatment ODI decrease was significant in the non-PSS group only (*p* = 0.014 and 0.021). The intergroup differences in ODI were not significant before treatment (*p* = 0.25), as well as at three months after treatment (*p* = 0.085). The difference six months after treatment was more expressed (*p* = 0.036) in the non-PSS group.

#### 3.2.4. Adverse Effects

No serious treatment-related complication was observed. Two patients in the non-PSS group presented with transient early post-treatment VAS increase (from 5 to 6 and from 8 to 10 points, respectively).

## 4. Discussion

The rationale for this study is to elucidate the potential benefits of minimally invasive PRF–DRG procedure in the treatment of CLRP in subjects who either have or have not undergone previous lumbar spine surgery. It is known that minimally invasive PRF–DRG treatment can be performed repeatedly [20]. In this regard, the aim was to verify whether a single PRF–DRG procedure can effective relief CLRP in the compared groups of patients. 

The results of this study indicate that a single PRF–DRG procedure has significantly lower efficacy in the treatment of L5 or S1 CLRP in patients with a history of previous lumbar spine surgery. The retrospective study of PRF–DRG treatment of CLRP by Abejón et al. [20] involving 54 patients showed similar results: a significant decrease of pain in the numerical rating scale (NRS) and global perceived effect (GPE) was observed in patients with disk herniation or spinal stenosis but not in those with FBSS. A more recent retrospective study by Yang et al. [21] evaluating the efficacy of PRF–DRG combined with transforaminal epidural steroid injection (0.2% Ropivacaine with 2.5 mg Betamethasone) in 34 patients with disk herniation, spinal stenosis, or FBSS showed significant relief of CLRP (measured by VAS) in all three groups, including FBSS. The difference in the outcomes between both cited studies could possibly be explained by the augmentation of PRF–DRG treatment by using locally injected medications. Moreover, these studies showed differences in PRF activity duration: 3 × 240 s (Yang et al.) versus 1 × 120 s (Abejón et al.); some patients underwent PRF–DRG procedures repeatedly or were treated at more than one radicular level. Furthermore, the study by Yang et al. was not specifically focused on L5 or S1 levels. Another retrospective trial studying the combination of PRF–DRG procedure and transforaminal epidural steroid injection (0.5% Lidocaine with 5 mg Dexamethasone) in the treatment of CLRP was conducted by Kim et al. [22]. This study included a total of 60 patients. Good analgesic treatment result was defined as ≥50% reduction of the pain score on day 30. This was achieved in 28.6% of PSS patients, which was not significantly different from the non-PSS group. A multicentric prospective trial by Van Boxem et al. [8] studying PRF–DRG treatment efficacy included 65 patients with CLRP (23.1% of them with FBSS). The authors reported overall clinical success, namely reduction of NRS ≥ 2 points or GPE of 1 or 2 (fully recovered or much improved) in 55.4% of patients at 6 months after treatment. However, this study focused on the treatment of L5 or S1 CLRP and did not specify the outcome of FBSS patients. In a triple-blinded placebo-controlled study by Shanthanna et al. [23] focused on PRF–DRG treatment efficacy in CLRP, a total of 31 randomized patients were enrolled. Among these, four patients with previous spine surgery were enrolled in the PRF group, and two patients in the placebo group. The differences in mean VAS between the PRF group and placebo group were not significant at four weeks or at three months after the treatment; the differences in mean ODI scores were not significant either.

It is not likely that the intergroup difference in BMI could significantly bias the study results. Both overweight and obesity are known risk factors for lumbar radicular pain and sciatica [24]. Instead, the same or even somewhat increased PRF–DRG treatment efficacy might have been expected in the non-PSS group if the BMI values were lower.

Based on the available literature knowledge, PRF–DRG can be considered a safe procedure [25]. Nevertheless, even in the case of adequate fluoroscopy or CT navigation control, the possibility of mechanical injury or hematoma formation resulting from needle manipulation cannot be completely eliminated. Gabrhelik et al. [26] mentioned a rare procedure-related mechanical spinal cord injury in a patient with severe scoliosis.

Nowadays, both CRF and PRF are used to treat a variety of painful conditions, including spinal and joint pain. PRF can also be used to reduce pain when peripheral nerve structures are affected (e.g., postherpetic or occipital neuralgia) [27]. In the last few decades, since the PRF began to be used in pain management, a number of papers evaluating its effectiveness have been published. Recent systematic review and meta-analysis by Farì et al. aimed to evaluate the efficacy of RF in treating musculoskeletal pain. Overall, 26 randomized controlled trials (RCT) were included—fifteen RCTs regarding the spine RF, five RCTs regarding the knee RF, three RCTs regarding the SI joint RF, and three RCTs regarding the shoulder RF. The outcome measures were pain, disability, and quality of life. With regard to the spine, the article mentioned, in particular, pain treatment utilizing CRF. However, within the set criteria, the PRF seemed to be effective in alleviating cervical radicular pain [28]. 

Vanneste et al. discussed PRF treatment of chronic pain in a review encompassing 121 publications, 37 of them concerning radicular pain themes. The authors found that non-neurological complications associated with PRF have been reported in more than 200 publications and further that PRF–DRG should be considered for the treatment of radicular pain. The authors highlight the need for high-quality RCTs and the identification of optimal parameters for the application of PRF in clinical practice in future research [25].

Facchini et al. evaluated the efficacy of PRF treatment of pain associated with different spinal conditions in a comprehensive review. Among others, four RCTs evaluating the efficacy of PRF–DRG on cervical radicular pain and three RCTs for PRF–DRG treatment of lumbosacral radicular pain were included. The authors concluded that from the available evidence, the use of PRF–DRG in cervical radicular pain is compelling; with regard to its lumbosacral counterpart, the use of PRF cannot be similarly advocated in view of the absence of standardization of PRF parameters, enrolment criteria, and different methods in reporting results; but the evidence is interesting [14].

We are aware of certain limitations of our study. The retrospective study design and a relatively small number of patients belong to the general limitations. However, we believe that the patient sample size is comparable to the cited studies [20,21,22,23]. Shanthanna et al. state that a large-scale clinical trial to establish the PRF–DRG treatment efficacy is not practically feasible considering the small effect size, which would necessitate the recruitment of a challengingly large number of participants over a number of years [23]. Etiological heterogeneities of CLRP and the number of previous lumbar spine surgical procedures in the patients within the PSS group represented more specific limitations and were not specifically considered. A larger cohort of patients would be appropriate to draw statistically powered conclusions about the impact of individual spine surgeries on the outcome of PRF–DRG treatment. Sluijter, in his paper evaluating the role of RF in FBSS, states that patients with FBSS are not uniform, and because the origin of the pain is commonly multifocal, many patients undergo a number of RF procedures within a relatively short period. This is a problem of evaluating the results of RF for spinal pain, and it is even more applicable to FBSS [29]. Possible effects of lifestyle habits (e.g., smoking, physical activity) were not studied, albeit these factors may modify the course of CLRP [30]. Although CLRP was the leading symptom in each patient, additional minor contributing factors related to it, e.g., facet joint pain, could not be completely ruled out. Nordstoga et al. [31] suggest that the presence of additional musculoskeletal pain may limit the success rate of the radicular pain treatment. Kim et al. [22] state that comorbid musculoskeletal pain and previous epidural injection response appear to affect the outcome of PRF–DRG treatment in patients with CLRP. From this point of view, the presented study could be criticized for not performing a diagnostic blockade of the DRG with a local anesthetic. However, there is still some controversy about this approach. Lee et al. [32] state that diagnostic blockade performed before PRF–DRG treatment has no significant effect on patient satisfaction, pain scores, or medication reduction. In addition, the application of a diagnostic block increases the cost of treatment. Thus, PRF–DRG procedure without screening DRG blockade is considered less invasive and more cost-effective. Eventually, the efficacy of PRF–DRG treatment was not evaluated in shorter (e.g., one-month) time intervals. Hence, possible beneficial short-term effects of the PRF–DRG procedure cannot be commented upon.

## 5. Conclusions

The results of this retrospective study indicate that a single PRF–DRG procedure is significantly less effective in the treatment of unilateral CLRP (at L5 or S1 level) in patients with a history of previous lumbar spine surgery when compared to the subjects without previous surgery. Such observation can aid in the decision-making when PRF–DRG treatment is considered in subjects with CLRP. Further research on this topic will be needed to draw more detailed conclusions.

## Figures and Tables

**Table 1:** Patients’ demographic characteristics.

	Non-PSS	PSS	*p*-Value
*n*	30	20	
age (median/q3-q1)	57.5/51.8–66.2	53.5/46.5–61	0.25
sex (male/female)	12/18	9/11	0.78
BMI (median/q3-q1)	29.2/26.6–33.4	26.5/24.4–29	0.043
treated DRG (L5/S1)	15/15	14/6	0.11
smokers *n* (%)	3 (10%)	2 (10%)	

The data are presented as a number (percent) unless otherwise noted. Age and body mass index (BMI) values are presented as the median/interquartile range (q3-q1)—Non-PSS and PSS groups of patients without and with a history of previous lumbar spine surgery.

**Table 2:** Characteristics of etiology and previous spine interventions within the PSS group.

Interventions	Total	Non-Fusion	Fusion	Disc Replacement
*n*	20	16	3	1
Etiology				
trauma	1	0	1	0
disc hernia	14	14	0	0
foraminostenosis	8	5	2	1
instability	3	0	3	0
Spinal segments				
L4-5/L5-S1	18	9/7	1/0	0/1
>1 spinal segment	2	0	1	1
Repeat surgery	1	1	0	0

The data are presented as a number. PSS, previous spine surgery.

## Data Availability

The data presented in this study are available on request from the corresponding author.
